# Spatia: Multimodal Model for Prediction and Generation of Spatial Cell Phenotypes

**Published:** 2025-07-07

**Authors:** Zhenglun Kong, Mufan Qiu, John Boesen, Xiang Lin, Sukwon Yun, Tianlong Chen, Manolis Kellis, Marinka Zitnik

**Affiliations:** 1 Harvard University; 2 UNC-Chapel Hill; 3 MIT

## Abstract

Understanding how cellular morphology, gene expression, and spatial organization jointly shape tissue function is a central challenge in biology. Image-based spatial transcriptomics technologies now provide high-resolution measurements of cell images and gene expression profiles, but machine learning methods typically analyze these modalities in isolation or at limited resolution. We address the problem of learning unified, spatially aware representations that integrate cell morphology, gene expression, and spatial context across biological scales. This requires models that can operate at single-cell resolution, reason across spatial neighborhoods, and generalize to whole-slide tissue organization. Here, we introduce Spatia, a multi-scale generative and predictive model for spatial transcriptomics. Spatia learns cell-level embeddings by fusing image-derived morphological tokens and transcriptomic vector tokens using cross-attention and then aggregates them at niche and tissue levels using transformer modules to capture spatial dependencies. Spatia incorporates token merging in its generative diffusion decoder to synthesize high-resolution cell images conditioned on gene expression. We assembled a multi-scale dataset consisting of 17 million cell-gene pairs, 1 million niche-gene pairs, and 10, 000 tissue-gene pairs across 49 donors, 17 tissue types, and 12 disease states. We benchmark Spatia against 13 existing models across 12 individual tasks, which span several categories including cell annotation, cell clustering, gene imputation, cross-modal prediction, and image generation. Spatia achieves improved performance over all baselines and generates realistic cell morphologies that reflect transcriptomic perturbations.

## 1 Introduction

Understanding the interplay between cellular morphology, gene expression, and spatial organization is essential for modeling tissue function and cell states in health and disease [56, 55]. Image-based spatial transcriptomic (ST) technologies enable high-resolution profiling of gene expression in intact tissue, along with matched cellular morphology derived from microscopy images [54, 7, 26, 36]. However, existing machine learning approaches often analyze morphology and gene expression separately, which limits their ability to learn representations of cellular phenotypes within spatial context. The central challenge is to learn unified representations that (i) capture the joint structure between image and gene modalities [5, 40], (ii) preserve spatial dependencies at the single-cell level [2, 65], and (iii) generalize across scales from local niches to whole-slide tissue context [49]. Naive fusion strategies, such as concatenating gene expression vectors with image features or training separate unimodal models, have limited ability to capture nonlinear and context-dependent relationships between modalities [35]. These limitations are amplified in spatial omics, where cellular identity and state are determined not only by intrinsic features but also by neighboring cells and broader tissue architecture. Existing models fall short in integrating spatial, molecular, and morphological information at single-cell resolution. Single-cell foundation models focus on transcriptomics and ignore morphology entirely [11, 28] or focus on spot-level spatial correlations [58, 59, 65, 49, 34]. Pathology models [8, 9] excel at whole-slide image analysis but disregard molecular information. Vision-language models [25, 38, 14] rely on textual supervision and cannot model image-gene relationships or spatial dependencies. Recent multimodal ST models [37, 6] aim to align histology with transcriptomics, but operate only at patch or spot resolution and lack single-cell granularity. As highlighted in recent evaluations of multimodal LLMs for vision-language reasoning [25, 39], these models struggle with grounding in spatial structure, compositional reasoning, and fine-grained biological semantics.

These limitations span three key dimensions. First, they fail to capture the full range of morphological variation and gene expression patterns at single-cell resolution, which is essential for understanding cell identity, state, and function. Second, they do not model spatial interactions across scales. Biological processes are governed not only by individual cell properties but also by local neighborhoods (niches) and tissue-level organization. Capturing these dependencies requires models that integrate cell-intrinsic features with context-aware representations across multiple spatial levels. Third, most models cannot learn from paired image-gene measurements, and therefore lack the capacity for bidirectional inference, such as predicting expression from morphology or generating cell images from transcriptomic profiles. Moreover, vision-language models lack the inductive biases necessary for modeling subcellular structure. They also do not support efficient generation conditioned on dense molecular inputs. In contrast, spatial cell phenotypes require generating detailed cell images that reflect transcriptomic perturbations while preserving morphological realism. This demands scalable generative models that can synthesize cell-level morphology based on gene expression, support conditional editing, and operate at the scale of millions of cells.

### Present work.

**Figure 1:**
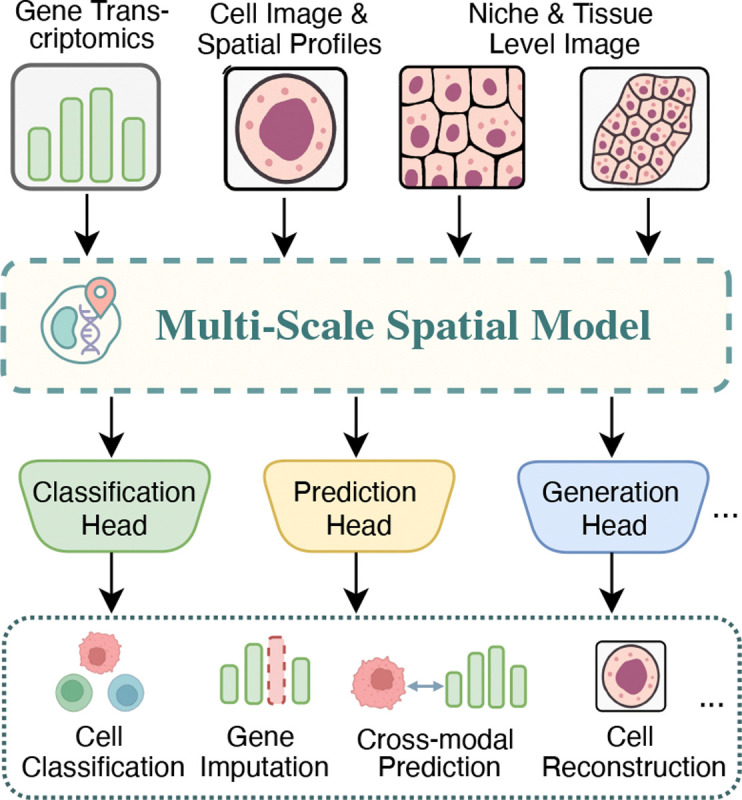
Spatia is a multi-scale spatial model for predictive and generative tasks.

We introduce Spatia, a multi-level model for generative and predictive modeling of spatial cell phenotypes (Fig. 1). Spatia integrates cell morphology, gene expression, and spatial coordinates within a unified model. The model consists of three components. At the *cellular level*, Spatia fuses image-derived morphological tokens and transcriptomic embeddings using cross-attention to produce a single-cell representation that captures both visual and molecular features. At the *niche level*, Spatia groups neighboring cells into spatial patches (e.g., 256×256 pixels) and applies a transformer to model local cell-cell interactions. At the *tissue level*, a global transformer aggregates niche representations to capture long-range dependencies across the full slide. Spatia is trained on MIST (Multi-scale dataset for Image-based Spatial Transcriptomics), a newly assembled multi-level dataset of image-based spatial transcriptomics. MIST (MIST-C-17M, MIST-N-1M, MIST-T-10K) contains 17 million cell-gene pairs, 1 million niche-gene pairs, and 10,000 tissue-gene pairs, spanning 49 donors, 17 tissue types, and 12 disease contexts. Each instance links morphology and gene expression at matched spatial scales, enabling fine-grained multimodal representation learning. Spatia supports both predictive tasks (e.g., classification, regression, spatial inference) and generative tasks (e.g., synthesizing cell images from gene expression). To improve inference efficiency, we introduce a token merging mechanism into the generative diffusion decoder, guided by classifier-free attention scores to retain important tokens while reducing compute.

Our main contributions include: ① **Joint modeling of cell morphology and gene expression** We introduce a cross-attention fusion mechanism that aligns morphological tokens with transcriptomic embeddings at the single-cell level. This alignment yields unified embeddings that preserve modality-specific detail while capturing their contextual relationships. ② **Multi-level spatial context modeling**. We develop a hierarchical transformer architecture that encodes spatial dependencies at the cell, niche, and tissue levels. This design enables modeling of both local cell-cell interactions and long-range tissue organization within a single unified model. ③ **Token-guided conditional image synthesis**. We develop an efficient conditional diffusion module to synthesize a new cell image $C_{i}^{'}$ i that reflects the morphological changes of gene perturbation. A training-free token merging method is applied to accelerate the generation of high-resolution cell images while reducing computational overhead. ④ **Bidirectional inference between form and function**. We train Spatia using paired image-expression data with cross-modal reconstruction losses and conditional generation objectives. This setup enables bidirectional inference: predicting gene expression from morphology and generating cell images from transcriptomic profiles. ⑤ **New benchmarking dataset**. We construct and release MIST, a new dataset for spatial transcriptomics containing 17 million cell-gene, 1 million niche-gene, and 10, 000 tissue-gene pairs across 49 donors, 17 tissue types, and 12 disease contexts, with one-to-one mappings between morphology and transcriptomic profiles. ⑥ **Evaluation across modalities and scales**. We benchmark Spatia against 13 existing models across 12 individual tasks, which span several categories including cell annotation, cell clustering, gene imputation, cross-modal prediction, and image generation. Spatia outperforms all baselines across scales and modalities.

## 2 Related Work

### Single Cell Models.

Foundation models for single-cell (non-spatial) transcriptomics have rapidly advanced, leveraging large-scale pretraining to support diverse downstream tasks such as cell type annotation, gene network inference, and perturbation prediction [11, 67]. Notable models include scGPT [11], scBERT [67], scPRINT [28], scMulan [1], scFoundation [19], scInterpreter [31], scHyena [44], GET [16], SCimilarity [21], and xTrimoGene [18]. These models are pretrained on repositories encompassing tens to hundreds of millions of cells, allowing them to capture complex transcriptional grammars, gene regulatory networks, and cellular heterogeneity across diverse biological contexts [19, 16]. However, they focus on transcriptomic data, lacking integration with spatial or imaging modalities, which are crucial for understanding cellular context within tissues.

### Spatial Transcriptomics Models.

Recent models include scGPT-spatial [59], which continually pretrains an scRNA-seq foundation model on multiple platforms of spatial data; CellPLM [65], a transformer-based cell language model pretrained on spatially resolved transcriptomic data to encode inter-cell relations; Nicheformer [49], a multi-organism transformer pretrained on over 110 million dissociated and spatial cells spanning human and mouse tissues for spatial composition and region label prediction Nichecompass [2], which utilizes existing knowledge of inter- and intracellular interaction pathways to learn an interpretable latent space of cells across multiple tissue samples, etc [24, 43]. However, most methods operate at spot-level resolution [58, 68, 64], lack single-cell granularity, and neglect integration of high-resolution histology or full-slide spatial context.

### Computational Pathology Models.

Vision-only models like HIPT [8] and UNI [9] harness hierarchical and self-supervised ViT pretraining on gigapixel WSIs, achieving state-of-the-art performance in cancer subtyping and multi-task evaluations. Vision-language approaches such as CONCH [38] and TITAN [14] employ contrastive and generative alignment with captions and reports to enable retrieval and report generation. Multimodal image-omic models such as ST-Align [37], STimage-1K4M [6], HEST-1k [27], Hist2Cell [71], and Stem [72] integrate spatial transcriptomics and morphology for gene expression inference and cell mapping. However, existing models are also constrained to spot-level resolution and do not capture single-cell granularity, which is crucial for dissecting cellular heterogeneity and microenvironmental interactions. Vision-only models lack explicit neighborhood or multi-scale tissue context, while vision-language models depend heavily on textual annotations, which can vary in quality and are often unavailable in high-throughput spatial settings.

### Generative Models.

Diffusion-based generative models have been applied to a large range of tasks in different domains. A popular strategy among them is to formulate the task as a conditional generation problem [69], using diffusion models to predict the distribution of outputs conditioned on the inputs [22, 13]. This has achieved success in text-to-image generation [46, 62], image super-resolution [47], image inpainting [45], etc. It is also used in applications for different domains such as cellular morphology painting [41], Heartbeat Waveform Monitoring [63], and protein pocket generation [70], etc. Meanwhile, efforts have been made to make diffusion models more efficient and accurate. [33] reduces the computation of the image decoder via data distillation. Other works [51, 53, 52] reduce the required number of denoising steps. Training-free efficiency enhancement schemes are also explored [60, 29].

## 3 Problem Formulation

Spatial transcriptomics technologies provide unprecedented opportunities to study biological systems by capturing gene expression profiles while preserving spatial location within tissue samples. Recent advancements, particularly in image-based spatial transcriptomics, offer high-resolution data that includes both cellular morphology and gene expression at a single-cell level [26]. This presents a unique challenge and opportunity to develop computational frameworks that can effectively integrate these rich, multimodal data sources to gain a deeper understanding of cellular states and interactions within their native spatial context.

We consider learning a unified, multi-scale representations that integrate cellular morphology and gene expression from spatial transcriptomic (Fig. 1). We begin with a cell-level dataset:

(1) $${𝒟}_{cell}={\left\{\left(C_{i},g_{i},s_{i}\right)\right\}}_{i=1}^{M},$$

where $C_{i}\in R^{H\times W\times 3}$ is the high-resolution cropped image of cell $i,\;g_{i}\in R^{G}$ is its gene-expression vector of the cell, and $s_{i}=\left(x_{i},y_{i}\right)\in R^{2}$ denotes its spatial coordinate. We learn a single embedding $z_{i}^{c}$ for each cell i that captures its morphology, transcriptome, and spatial context in one coherent vector via a model:

(2) $$z_{i}^{c}={ℱ}_{\mathrm{cell}}\left(C_{i},g_{i},s_{i}\right)\in R^{D}$$

Next, we group cells into non-overlapping spatial *niches* of the slides with a corresponding set of cells ${𝒞}_{j}$. For niche j, we add the gene-expression vectors of the cells $g_{j}^{n}=\sum_{i\in {𝒞}_{j}}g_{i}$, and encode its larger image patch $P_{k}^{\mathrm{reg}}$ using a dedicated niche-level encoder ${ℱ}_{\mathrm{niche}}$. Similarly at the tissue level, we group cells into larger non-overlapping spatial regions of the slides with a corresponding set of cells ${𝒞}_{k}$. We pass the data into a global transformer ${ℱ}_{\mathrm{tissue}}$ to capture long-range dependencies across the entire slide. The niche and tissue level dataset and embeddings are:

(3) $$\left\{\begin{array}{l} {𝒟}_{\mathrm{niche}}={\left\{\left(N_{j},g_{j}^{n},s_{j}\right)\right\}}_{j=1}^{P},\\ {𝒟}_{\mathrm{tissue}}={\left\{\left(T_{k},g_{k}^{t},s_{k}\right)\right\}}_{k=1}^{Q} \end{array},\;\left\{\begin{array}{l} z_{j}^{n}={ℱ}_{\mathrm{niche}}\left(N_{j},g_{j}^{n},s_{j}\right),\\ z_{k}^{t}={ℱ}_{\mathrm{tissue}}\left(T_{k},g_{k}^{t},s_{k}\right) \end{array}\in R^{D},\right.\right.$$

To obtain the final unified embedding for each cell i, we attend from its cell-level representation to both the corresponding niche and the global tissue context:

(4) $$z_{i}={ℱ}_{\mathrm{fusion}}(\underset{\mathrm{cell}\;\mathrm{level}}{\underbrace{z_{i}^{c}}},\underset{\mathrm{niche}\;\mathrm{level}}{\underbrace{z_{j}^{n}}},\underset{\mathrm{tissue}\;\mathrm{level}}{\underbrace{z_{k}^{t}}})\in R^{D},$$

The resulting unified embedding $z_{i}$, along with the intermediate scale-specific embeddings $z_{i}^{c},\;z_{i}^{n}$, and $z_{i}^{t}$, can be leveraged for a wide range of downstream biomedical tasks. These include: (i) *cell type annotation*, by training an MLP classifier on $z_{i}$; (ii) *spatial identification*, through clustering or regional softmax over the embeddings; (iii) *gene expression imputation*, by learning a regression model that maps $z_{i}$ to its corresponding gene expression vector; (iv) *image generation*, using $z_{i}$ as a condition to synthesize realistic cell images via generative decoder; and (v) *composition prediction*, by estimating the cell type distribution within a niche based on the aggregated embeddings, etc.

## 4 Spatia Model

**Figure 2:**
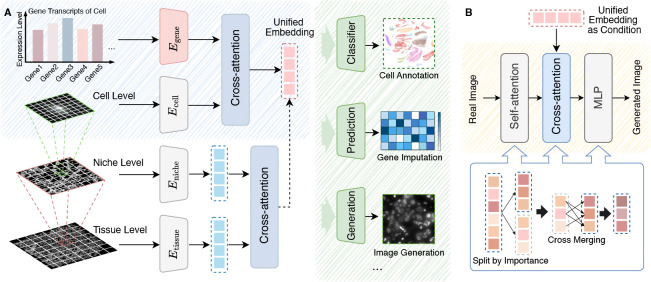
A) Overview of Spatia. B) Cell image generation with token merging.

As shown in Fig. 2A, Spatia is designed to learn comprehensive, multi-scale representations by integrating cellular morphology, gene expression, and spatial context from image-based spatial transcriptomics data. It operates hierarchically, first learning unified single-cell representations by fusing cell image and gene data, then refining these representations by modeling spatial relations within the tissue microenvironment.

### 4.1 Unified Single-Cell Representation Learning

We aim to generate a unified embedding for each cell that captures synergistic information from its morphology and gene expression profile. We employ separate encoders for the image and gene expression modalities to extract initial feature representations for each cell i.

### Morphological Feature Extraction.

We process $C_{i}$ using a vision transformer (ViT) based image encoder, $E_{\mathrm{cell}}$. Following standard ViT practice, the image patch is divided into a sequence of non-overlapping smaller patches (e.g., 64 × 64 pixels). These image patches are linearly projected into an embedding space and prepended with a learnable [CLS] token. The output of the cell encoder for cell i is a sequence of patch embeddings, forming the cell matrix.

### Gene Expression Feature Extraction.

The gene expression profile $g_{i}$ for cell i is typically a vector of gene counts or normalized expression values. We process $g_{i}$ using a gene encoder, $E_{\mathrm{gene}}$. We apply the pretrained scPRINT [28] as the gene backbone to embed the gene expressions. For a panel of $N_{\mathrm{gene}}$ genes, we obtain an embedding matrix $x_{i}\in R^{N_{\mathrm{gene}}\times D}$, where each row corresponds to a specific gene’s expression mapped into the latent space, potentially using learnable gene-specific embeddings modulated by expression values. The modality-specific encoders are defined as:

(5) $$X_{i}^{c}=E_{\mathrm{cell}}\left(C_{i}\right)\in R^{N_{\mathrm{patch}}\times D},\;x_{i}^{g}=E_{\mathrm{gene}}\left(g_{i}\right)\in R^{D},$$

where $N_{\mathrm{patch}}$ is the number of patches extracted from $C_{i}$. $X_{i}^{c}\in R^{N_{\mathrm{patches}}\times D}$ is the cell matrix.

### Multimodal Feature Fusion.

To integrate information from both modalities at the single-cell level, we employ a cross-attention mechanism, as depicted in Fig. 2. Specifically, we use the cell matrix $X_{i}^{c}$ as the query sequence and the gene matrix $x_{i}^{g}$ as the key and value sequences. The fusion module then produces the embedding $z_{i}^{c}=CrossAttn\left(Q=X_{i}^{c},K=x_{i}^{g},V=x_{i}^{g}\right)$, which aligns fine-grained morphological tokens with the transcriptomic tokens to obtain the single, unified representation for cell i, that encapsulates fused morphological and transcriptomic information.

### 4.2 Hierarchical Spatial Integration via Multi-Level Transformers

Building upon the unified cell representations derived earlier, Spatia employs a multi-level learning approach with dedicated transformer modules to integrate spatial context and learn representations at progressively larger scales: the niche level and tissue (slide) level. This approach enables the model to capture cellular interactions within neighboring niches as well as in the global tissue area. Moreover, it allows the cell representation to obtain spatial-aware relational information.

#### 4.2.1 Niche Representation Learning

##### Niche Definition.

**Figure 3:**
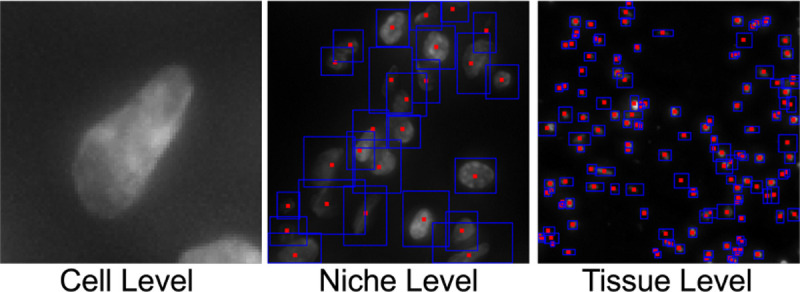
Three levels of whole slide image.

We define an area of space containing neighboring cells as a niche, as shown in Fig. 3. This typically characterizes local tissue structures, such as tumor niches or immune niches, and they differ not only in gene expression patterns but also in the composition of cell types.

Inside each niche, we align the cell ID with each cell and also record its gene expression. These niches also have spatially dependent labels, which are obtained by integrating similarities in gene expression profiles and spatial proximity. By incorporating both molecular and spatial context, niche-level features capture the microenvironmental heterogeneity that is critical for understanding cellular interactions and functional roles within tissues.

##### Niche Feature Extraction and Fusion.

Given a pre-defined niche image $N_{j}$ and cell set $\left\{c_{i}|c_{i}\in {𝒞}_{j}\right\}$, we first extract a sequence of morphological tokens $X_{j}^{n}=E_{\mathrm{niche}}\left(N_{j}\right)\in R^{N_{\mathrm{patch}}\times D}$, where $E_{\mathrm{niche}}$ is a ViT-based encoder and $N_{\mathrm{patch}}$ is the number of local image patches. In parallel, we gather the unified per-cell embeddings ${\left\{z_{j}^{c}\right\}}_{j\in {𝒞}_{j}}$ and compute their pooled context ${\bar{z}}_{k}=\frac{1}{\left|{𝒞}_{j}\right|}\sum_{i\in {𝒞}_{j}}z_{i}^{c}\in R^{D}$. We then fuse morphology and cellular context via cross-attention:

(6) $$z_{j}^{n}=CrossAttn\left(Q={\bar{z}}_{k},K=X_{j}^{n},V=X_{j}^{n}\right)\in R^{D}.$$

The neighbouring morphology tokens $X_{k}^{n}$ serve as the query sequence, while the niche cell context vector ${\bar{z}}_{k}$ serves as the key and value. The niche representation $z_{j}^{n}\in R^{D}$ captures how individual cells interact with their immediate neighbors, encoding meaningful micro-scale spatial relationships.

#### 4.2.2 Tissue Representation Learning

At the tissue (slide) level, the transformers aggregate information from multiple niches. By processing a larger spatial extent, the model can capture global patterns and long-range interactions within the tissue, providing a more comprehensive understanding of the tissue-wide spatial context.

Similar as the previous level, we aggregate the learned niche representations ${\left\{n_{k}\right\}}_{k=1}^{N_{\mathrm{regions}}}$ using a tissue encoder ($E_{\mathrm{tissue}}$) to get $X_{k}^{t}$. The input sequence consists of the set of all niche representations ${\{n}_{k}\}$ obtained from the previous level. Positional information encoding the location of each region $R_{k}$ within the overall tissue grid is added. This can be achieved using learnable or fixed 2D positional embeddings $pe_{k}$ corresponding to the grid coordinates of region k. We attend the niche-level representation to both the corresponding cell and niche context:

(7) $$z_{j}^{t}=CrossAttn(Q=z_{j}^{n},\;K=X_{k}^{t},\;V=X_{k}^{t})\in R^{D},$$

Note that the embeddings from the encoders at each level can be used independently, and the fusion process can also vary depending on the specific downstream task scenario. Our approach also differs from multi-instance learning [17], where a “bag” of instances is typically summarized using a pooling operation to predict a single bag-level label, without explicitly modeling interactions among instances or their spatial arrangement. In contrast, Spatia explicitly models spatial relationships and integrates information across multiple scales, from single cells to niches, and finally to the slide level, leveraging the strengths of transformers for context aggregation at each level.

### 4.3 Self-Supervised Training Objectives

We train the Spatia using self-supervised objectives with paired multimodal data. This avoids reliance on potentially scarce manual annotations and allows the model to learn rich, biologically meaningful representations. We enforce consistency between the modalities and learn the fusion process by reconstructing the original data from the unified embedding via a dual decoder architecture.

#### Image Reconstruction.

An image decoder $D_{\mathrm{cell}}$ takes the unified embedding $z_{i}^{c}$ as input and aims to reconstruct the original cell image patch ${\overset{ˆ}{C}}_{i}=D_{\mathrm{cell}}\left(z_{i}^{c}\right)$. The reconstruction loss ${ℒ}_{\mathrm{img\_recon}}$ is typically the MAE [20] between the reconstructed and original image pixels.


(8) $${ℒ}_{\mathrm{img\_recon}}=\frac{1}{N}\sum_{i=1}^{N}{\left\|{\overset{ˆ}{C}}_{i}-C_{i}\right\|}_{2}^{2}$$

#### Gene Reconstruction.

A gene decoder $D_{\mathrm{gene}}$ takes the unified embedding $z_{i}^{c}$ (or $z_{j}^{n},\;z_{k}^{t}$) and aims to reconstruct the original gene expression profile $g_{i}$. Using a set of learnable gene query embeddings ${\left\{q_{g}\right\}}_{g=1}^{N_{\mathrm{gene}}}$, the decoder can attend to the unified cell embedding $z_{i}^{c}$ (acting as memory) to predict the gene embeddings ${\overset{ˆ}{g}}_{i}=D_{\mathrm{gene}}\left(z_{i}^{c}\right)$. We use masked language modeling [12] for the reconstruction loss. The overall loss function combines the selected objectives ${ℒ}_{\mathrm{total}}=\lambda_{\mathrm{cell}}{ℒ}_{\mathrm{img\_recon}}+\lambda_{\mathrm{gene}}{ℒ}_{\mathrm{gene\_recon}}$, weighted by hyperparameters $\lambda_{\mathrm{cell}}$ and $\lambda_{\mathrm{gene}}$.

### 4.4 Accelerated Cross-Modal Conditional Generation

Gene expression changes, such as overexpression, knockdown, or knockout, can induce alterations in cell shape and texture [4]. To assess the generative capability of Spatia, we introduce a conditional diffusion decoder to synthesize realistic cell images from the fused embeddings, enabling us to visualize these effects.

Given an original cell-gene pair with coordinate ($C_{i},\;g_{i},\;s_{i}$) and a modified gene vector $g_{i}^{\prime }$, Spatia synthesizes a new cell image $C_{i}^{\prime }$ that reflects the downstream morphological effects of the gene perturbation. We continue to utilize the unified cell embedding *z*_*i*_ as the conditioning signal, denoted as $z_{i,\mathrm{cond}}$. We employ a frozen variational autoencoder to map between the pixel space and a compact latent space. Additionally, a lightweight U-Net is used for denoising the latents. We define

(9) $$z_{i,\mathrm{cond}}=\left\{\begin{array}{ll} F_{\mathrm{fusion}}\left(C_{i},g_{i},s_{i}\right), & \mathrm{(training)}\\ F_{\mathrm{fusion}}\left(C_{i},g_{i}^{\prime },s_{i}\right), & \mathrm{(inference)} \end{array}\right.$$

at each attention layer. This process preserves original morphology anchored by $C_{i}$, while editing the shape and textures according to the new gene profile $g_{i}^{\prime }$.

To accelerate image synthesis during the inference phase, we utilize token merging in the attention layers of the diffusion model. Existing token merging methods [3] overlook the importance of individual tokens, potentially leading to the quality degradation of the synthesized images. These methods typically adopt exclusive pairings, where once a token is assigned to a particular group, it cannot be reassigned to other groups for merging. This constraint limits the model’s representational power, since a single token might be relevant to multiple groups concurrently. To tackle these problems, we first partition the token sequence of length N based on token importance, using classifier-free guidance (CFG) [23] as an importance indicator. CFG is designed to guide the predicted noise in a direction that better aligns with the given conditions, enabling a more effective assessment of token significance:

(10) $${\tilde{\epsilon }}_{\theta }\left(x_{t},t\right)=\epsilon_{\theta }\left(x_{t},t\right)+w\cdot \left(\epsilon_{\theta }\left(x_{t}|z_{i,\mathrm{cond}},t\right)-\epsilon_{\theta }\left(x_{t},t\right)\right),$$

where $\epsilon_{\theta }\left(x_{t}|y,t\right)$ is the noise prediction conditioned on $z_{i,\mathrm{cond}},\;\epsilon_{\theta }\left(x_{t},t\right)$ is the unconditional noise prediction, and w is the weight. We calculate its importance as the absolute value of its CFG score:

(11) $$I=\left|\epsilon_{\theta }\left(x_{t}|y,t\right)-\epsilon_{\theta }\left(x_{t},t\right)\right|,$$

We select a subset consisting of M tokens with the highest scores as *anchor tokens*. These anchor tokens serve as the primary elements to be preserved. Then, we measure pairwise token similarities using the query-key mechanism of the attention module. Given that these similarity measurements reflect the relationships among tokens, we construct a mapping operation to merge the tokens into the anchor tokens. Detailed processes of training and inference are provided in Appendix C.

## 5 Experiments

### 5.1 Datasets and Experimental Setup

#### MIST Datasets.

MIST comprises three nested scales: 1) **MIST-C**: 17M single cell-gene pairs; 2) **MIST-N**: 1M niche-gene pairs; 3) **MIST-T**: 10K tissue-gene entries. These splits enable precise mapping of cell morphology to transcriptomics at cellular, regional, and whole-slide levels, supporting multimodal representation learning across diverse biological contexts. We first load the full-resolution tissue image (0.2125*μ*m/px) and compute a maximum-intensity projection over z. The resulting 2D image is normalized to 8-bit [0, 255]. We use the cell boundary file to extract individual cell images. For each cell, we compute the minimal square region that fully contains the cell and crops the image accordingly. Each cell-gene example consists of this uint8 image patch and the corresponding per-cell transcript vector for a single gene, serialized into LMDB for efficient training (MIST-C). To form MIST-N, we tile each slide into a grid of non-overlapping 256 × 256 px niches, assign cells to their containing patch, and pool gene vectors within each niche. Each niche entry therefore includes the regional image patch and its aggregated gene profile. Finally, MIST-T summarizes each slide by its set of niche embeddings and positional metadata, with a size of 1024 × 1024, enabling tissue-level tasks such as global composition prediction and cross-slide transfer. All three scales are provided with consistent preprocessing, enabling end-to-end evaluation of cell-, niche-, and tissue-level tasks for machine learning research. The full dataset statistics are present in Appendix A.

#### Baselines.

We benchmark Spatia against a total of 13 models, including pathology models-CTransPath [61], GigaPath [66], CONCH [39], UNI [9], UNIv1.5 [9], H-Optimus-0 [48], Hibou [42], and PLIP [25]-as well as single-cell models: Geneformer [57], scGPT [11], CellTypist [15], scBERT [67], and CellPLM [67].

#### Experimental Setup and Implementation.

We evaluate Spatia across four groups of tasks: cell annotation, clustering, gene expression prediction, and biomarker status prediction. Additionally, we conduct studies to assess the multimodal and multi-scale capabilities of Spatia: (i) cross-modal embedding prediction (gene ↔ cell), (ii) conditional image generation from gene embeddings. Detailed information on the data, analysis, and results is presented in the following subsections, according to the corresponding research questions. Additional training settings and model configurations are provided in Appendix B.

### 5.2 R1: Cell Annotation & Clustering Tasks

We follow the settings in [65] and use Multiple Sclerosis (MS) dataset [50] to evaluate cell annotation performance and scRNAseq data [32]. Results are shown in Tab. 1. We report F1 and Precision scores for annotation task; ARI and NMI scores for clustering task. These results highlight Spatia’s ability on supervised and unsupervised single-cell analysis tasks compared to existing methods.

### 5.3 R2: Biomarker Status Prediction Tasks

Table 2 compares SPATIA against six models on invasive breast cancer dataset, evaluating ER, PR, and HER2 status from WSIs in the BCNB dataset. We follow the settings in [8] to embed the data into existing pathology models using modality-specific encoders. For example, we implement the image patches using a pretrained model (e.g., CONCH) and the expression data using a 3-layer MLP.

The modality-specific embeddings are then aligned using a contrastive objective, i.e., InfoNCE loss, by fine-tuning the image encoder and training the expression encoder from scratch. SPATIA consistently achieves the highest AUC and balanced accuracy across all three markers.

1. SPATIA attains an AUC of 0.902 and balanced accuracy of 0.785 for ER, improving over the prior best UNI by +0.011 AUC and +0.010 Bal.acc.
2. Our model reaches AUC 0.825 and Bal.acc 0.731, compared to UNI’s 0.820 AUC and 0.712 Bal.acc, a gain of +0.005 AUC and +0.019 Bal.acc.
3. HER2: SPATIA records AUC 0.744 and Bal.acc 0.643, outperforming UNI by +0.012 AUC and +0.002 Bal.acc. These demonstrate that integrating multi-scale spatial context with cross-modal embeddings yields enhanced capacity to capture morphological and molecular signals.

### 5.4 R3: Gene Expression Prediction Tasks

**Figure 4:**
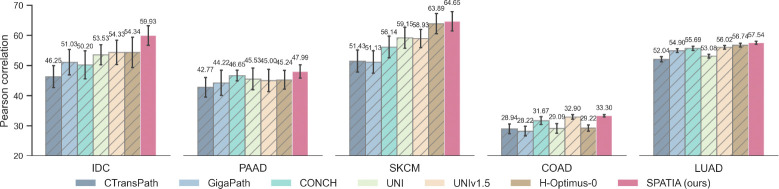
Gene expression prediction.

We use HEST-Bench [8] to evaluate Spatia for gene expression prediction task. Figure 4 reports Pearson correlation coefficients (PCC) for the top 50 highly variable genes on five cancer cohorts (IDC, PAAD, SKCM, COAD, LUAD).

We train a regression model to map model-specific patch embeddings to the log1p-normalized expression of the top 50 highly variable genes. We use XGBoost [10] regression model with 100 estimators and a maximum depth of 3. These consistent gains across diverse tissue types demonstrate that Spatia yields embeddings that more accurately capture gene-image relationships than existing single or dual modal architectures.

### 5.5 Applications: Cross-Modal Prediction and Generation

#### Cross-modal prediction.

We train a MLP decoder to reconstruct held-out gene expression $g_{i}$ from cell embeddings $C_{i}$ and, conversely, to predict cell embeddings from gene expression inputs. Reconstruction quality is measured via Pearson or Spearman correlation between predicted ${\overset{ˆ}{g}}_{i}$ (or ${\overset{ˆ}{C}}_{i}$) and ground truth.

#### Conditional Image Generation.

**Figure 5:**
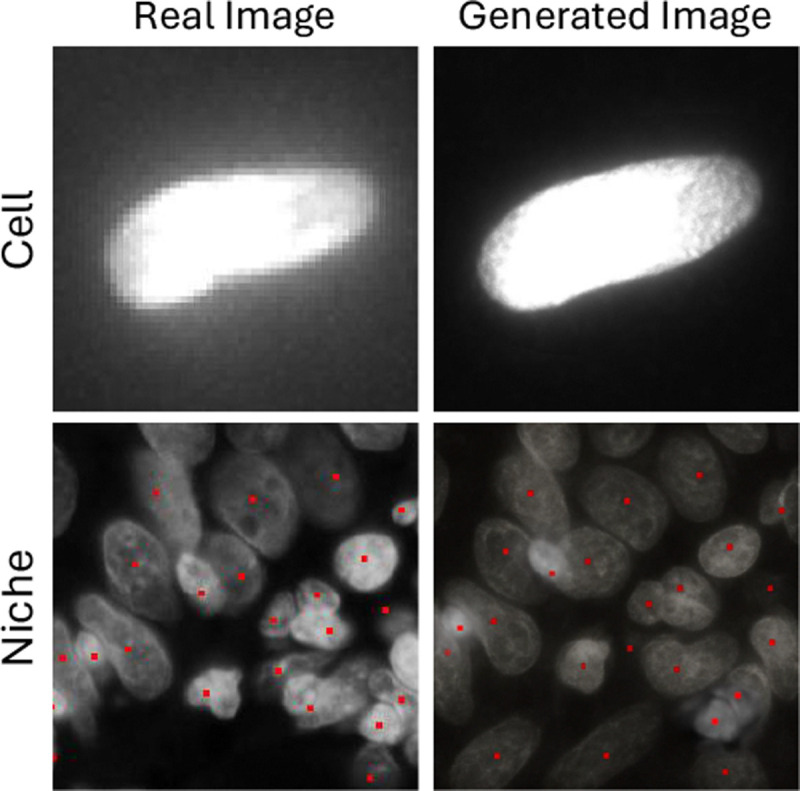
Image generation.

Using Spatia diffusion decoder, we generate synthetic cell images $C_{i}$ conditioned on gene embeddings to simulate perturbation effects. We evaluate image fidelity with PSNR and SSIM against original patches. Existing models (e.g. UNI, CONCH) lack image-generation capability and are marked N/A in Table 3. , Additionally, we present visualized examples in Fig. 5. The effectiveness of our token merging method during diffusion is further evaluated in Appendix C.

## 6 Ablation & Analysis

Table 4 reports an ablation on cell-type classification, starting from the cell-level backbone and incrementally adding: (i) reconstruction loss, (ii) multi-level hierarchy, (iii) cross-attention fusion. Adding the multi-level hierarchy produces the largest single improvement (0.27 loss ↓), underscoring the value of aggregation across scales. Collectively, these results show that each component meaningfully enhances our multi-scale representation.

## 7 Conclusion

Spatia is a multi-resolution model that integrates cellular morphology, spatial context, and gene expression for spatial transcriptomics. The model addresses a critical gap in existing approaches, which often treat these modalities in isolation and fail to capture the structured dependencies across biological scales. Spatia achieves strong performance on a range of generative and predictive benchmarks, including cell type classification, gene expression imputation, spatial clustering, and image synthesis. The hierarchical attention modules model local cell-cell interactions and long-range dependencies, and as we show, self-supervised objectives, including cross-modal reconstruction and diffusion-based generation, support robust, label-efficient training. The model is trained and evaluated on MIST, a large multi-scale dataset assembled from 49 image-based spatial transcriptomics samples across 17 tissue types and 12 disease contexts. MIST provides one-to-one mappings between image patches and transcriptomic profiles at single-cell, niche, and tissue levels. Spatia provides a foundation for modeling spatial omics with fine-grained resolution. Future work will explore extending the framework to additional spatial omics modalities, training on more single-cell data, integrating temporal dynamics, and scaling to larger cohorts for clinical applications.

## Figures and Tables

**Table 1:** Downstream tasks.

Method	Annotation		Method	Clustering	
	F1 (↑)	Precision (↑)		ARI (↑)	NMI (↑)

scGPT	0.703	0.729	PCA	0.843	0.812
CellPLM	0.709	0.702	CellPLM	0.867	0.823
scBERT	0.599	0.604	scGPT	0.856	0.828
CellTypist	0.667	0.693	Geneformer	0.461	0.586

Spatia	0.725	0.734	Spatia	0.870	0.831

**Table 2:** Logistic regression evaluation for ER/PR/HER2 status on BCNB.

Model	ER		PR		HER2	
	AUC	Bal.acc.	AUC	Bal.acc.	AUC	Bal.acc.

UNI	0.891	0.775	0.820	0.712	0.732	0.641
GigaPath	0.841	0.765	0.803	0.696	0.721	0.635
Hibou	0.832	0.754	0.801	0.694	0.705	0.630
CLIP	0.652	0.537	0.618	0.502	0.514	0.438
PLIP	0.712	0.603	0.695	0.587	0.611	0.524
CONCH	0.881	0.745	0.810	0.698	0.715	0.624

Spatia	**0.902**	**0.785**	**0.825**	**0.7309**	**0.744**	**0.643**

**Table 3:** Performance on cross-modal prediction and generation tasks.

Task	Cross modal Pred.		Cross modal Gen.	
	Pearson ↑	Spearman ↑	PSNR ↑	SSIM ↑

Spatia	0.43	0.41	24.80	0.65

**Table 4:** Ablation study

Method	Loss (↓)	Accuracy (↑)

Cell level only	0.405	0.93
+ MAE loss	0.396	0.94
+ Multi-level	0.369	0.97
+ Fusion	0.361	0.98

## A Dataset Statistics

Imaging-based spatial transcriptomics technologies allow us to explore spatial gene expression profiles at the cellular level. Xenium is a high-resolution, imaging-based in situ spatial profiling technology from 10x Genomics that allows for simultaneous expression analysis of RNA targets within the same tissue section. This assay can identify the location of target transcripts within the tissue, providing a single cell resolution map of expression patterns of all genes that are included in the selected probe panel. Our MIST dataset is assembled from 49 Xenium sources [26] spanning 17 tissue types, 49 donors, and 12 disease states, including: healthy (7) Breast cancer (5) Lung cancer (4) Adenocarcinoma (3) Ovarian cancer (2) cancer (melanoma) (2) Cervical cancer (1) Colorectal cancer (1) Invasive Ductal Carcinoma (1) Melanoma (1) acute lymphoid leukemia (1) cancer (1). The total dataset contains 17,515,676 total cells and 6,000 unique genes. Dataset Statistics is shown in Tab. 5 and Tab. 6.

**Table 5:** Xenium Datasets gathered from 10x Genomics (part 1).

Collection name	Tissue	Disease	Num. cells	Num. genes

Xenium_Preview_Human_Lung_Cancer_With_Add_on_2_FFPE	lung	cancer	531,165	392
Xenium_Preview_Human_Non_diseased_Lung_With_Add_on_FFPE	lung	healthy	295,883	392
Xenium_Prime_Breast_Cancer_FFPE	breast	cancer	699,110	5101
Xenium_Prime_Cervical_Cancer_FFPE	cervical	cancer	840,387	5101
Xenium_Prime_Human_Lung_Cancer_FFPE	lung	cancer	278,328	5001
Xenium_Prime_Human_Lymph_Node_Reactive_FFPE	lymph node	reactive hyperplasia	708,983	4624
Xenium_Prime_Human_Ovary_FF	ovary	adenocarcinoma	1,157,659	5001
Xenium_Prime_Human_Prostate_FFPE	prostate	adenocarcinoma	193,000	5006
Xenium_Prime_Human_Skin_FFPE	skin	melanoma	112,551	5006
Xenium_Prime_Ovarian_Cancer_FFPE	ovary	cancer	407,124	5101
Xenium_V1_FFPE_Human_Brain_Alzheimers_With_Addon	brain	alzheimers	44,955	354
Xenium_V1_FFPE_Human_Brain_Glioblastoma_With_Addon	brain	glioblastoma	40,887	319
Xenium_V1_FFPE_Human_Brain_Healthy_With_Addon	brain	healthy	24,406	319
Xenium_V1_FFPE_Human_Breast_IDC_Big_1	breast	invasive ductal carcinoma	892,966	280
Xenium_V1_FFPE_Human_Breast_IDC_Big_2	breast	invasive ductal carcinoma	885,523	280
Xenium_V1_FFPE_Human_Breast_IDC_With_Addon	breast	invasive ductal carcinoma	576,963	380
Xenium_V1_FFPE_Human_Breast_IDC	breast	invasive ductal carcinoma	574,852	280
Xenium_V1_FFPE_Human_Breast_ILC_With_Addon	breast	invasive lobular carcinoma	365,604	380
Xenium_V1_FFPE_Human_Breast_ILC	breast	invasive lobular carcinoma	356,746	280
Xenium_V1_Human_Brain_GBM_FFPE	brain	glioblastoma	816,769	480
Xenium_V1_Human_Colorectal_Cancer_Addon_FFPE	colorectal	cancer	388,175	480
Xenium_V1_Human_Ductal_Adenocarcinoma_FFPE	pancreas	ductal adenocarcinoma	235,099	380
Xenium_V1_Human_Lung_Cancer_Addon_FFPE	lung	cancer	161,000	480
Xenium_V1_Human_Lung_Cancer_FFPE	lung	cancer	278,659	289
Xenium_V1_Human_Ovarian_Cancer_Addon_FFPE	ovary	cancer	247,636	480
Xenium_V1_hBoneMarrow_acute_lymphoid_leukemia_section	bone marrow	acute lymphoid leukemia	225,906	477
Xenium_V1_hBoneMarrow_nondiseased_section	bone marrow	healthy	84,518	477
Xenium_V1_hBone_nondiseased_section	bone	healthy	33,801	477
Xenium_V1_hColon_Cancer_Add_on_FFPE	colon	cancer	587,115	425
Xenium_V1_hColon_Cancer_Base_FFPE	colon	cancer	647,524	325
Xenium_V1_hColon_Non_diseased_Add_on_FFPE	colon	healthy	275,822	425
Xenium_V1_hColon_Non_diseased_Base_FFPE	colon	healthy	270,984	325
Xenium_V1_hHeart_nondiseased_section_FFPE	heart	healthy	26,366	377
Xenium_V1_hKidney_cancer_section	kidney	cancer	56,510	377
Xenium_V1_hKidney_nondiseased_section	kidney	healthy	97,560	377

**Table 6:** Xenium Datasets gathered from 10x Genomics (part 2).

Collection name	Tissue	Disease	Num. cells	Num. genes

Xenium_V1_hLiver_cancer_section_FFPE	liver	cancer	162,628	474
Xenium_V1_hLiver_nondiseased_section_FFPE	liver	healthy	239,271	377
Xenium_V1_hLung_cancer_section	lung	cancer	150,365	377
Xenium_V1_hLymphNode_nondiseased_section	lymph node	healthy	377,985	377
Xenium_V1_hPancreas_Cancer_Add_on_FFPE	pancreas	cancer	190,965	474
Xenium_V1_hPancreas_nondiseased_section	pancreas	healthy	103,901	377
Xenium_V1_hSkin_Melanoma_Base_FFPE	skin	melanoma	106,980	282
Xenium_V1_hSkin_nondiseased_section_1_FFPE	skin	healthy	68,476	377
Xenium_V1_hSkin_nondiseased_section_2_FFPE	skin	healthy	90,106	377
Xenium_V1_hTonsil_follicular_lymphoid_hyperplasia_section_FFPE	tonsil	follicular lymphoid hyperplasia	864,388	377
Xenium_V1_hTonsil_reactive_follicular_hyperplasia_section_FFPE	tonsil	reactive follicular hyperplasia	1,349,620	377
Xenium_V1_humanLung_Cancer_FFPE	lung	cancer	162,254	377
Xenium_V1_human_Pancreas_FFPE	pancreas	cancer	140,702	377
Xeniumranger_V1_hSkin_Melanoma_Add_on_FFPE	skin	melanoma	87,499	382

## B Training & Implementation Details

### Training Details.

The hierarchical modules (${ℱ}_{\mathrm{cell}},\;{ℱ}_{\mathrm{niche}},\;{ℱ}_{\mathrm{tissue}}$) are trained concurrently with the corresponding dataset using primary reconstruction losses (MAE) computed on the corresponding embeddings ($z_{i}^{c},\;z_{i}^{n},\;z_{i}^{t}$). We use the AdamW optimizer [30] with a learning rate of 1e-3.

### Model Architecture.

Our model architecture, based on scPrint, has core components including: a GeneEncoder for processing gene expression data, which contains an embedding layer (Embedding) and a continuous value encoder (ContinuousValueEncoder); an image processing module based on ViTMAEForPreTraining, comprising a 12-layer ViT encoder (ViTMAEEncoder) and an 8-layer ViT decoder (ViTMAEDecoder); and an 8-layer FlashTransformerEncoder as the main sequence transformer.

The model also integrates multiple FusionLayers for multimodal feature fusion, an ExprDecoder for gene expression reconstruction, and multiple ClsDecoders for downstream classification tasks. Key hyperparameters are summarized in Tab. 7.

## C Image Generation Process

### C.1 Training & Implementation Details

We replace the pure reconstruction decoder of with a diffusion module that, given an original cell-gene pair ($C_{i},\;g_{i}$) and a modified gene vector $g_{i}^{\prime }$, synthesizes a new cell image reflecting the downstream morphological effects of the gene perturbation. We continue to use the cell embedding

$$z_{i,\mathrm{cond}\;}=F_{\mathrm{fusion}}\left(C_{i},g_{i},s_{i}\right)\in R^{D}$$

as our *conditioning signal*, since it compactly encodes morphology, transcriptomics, and spatial context. Biologically, gene expression changes-whether overexpression, knockdown, or knockout-can induce pronounced alterations in cell shape, subcellular structure, and texture. A conditional diffusion editor allows us to visualize these effects by editing a real cell image: the model preserves the original morphology, while introducing changes driven solely by the altered gene expression.

#### C.1.1 Module Structure

We adopt a frozen variational autoencoder ($E_{\mathrm{diff}},\;D_{\mathrm{diff}}$) (e.g. the Stable Diffusion VAE) to map between pixel space and a compact latent space. A lightweight U-Net (40M parameters) is used to denoise latents. Each down- and up-sampling block contains: 1) A convolutional ResBlock. 2) Self-attention over the spatial tokens 3) Cross-attention where the flattened tokens attend to $z_{i,\mathrm{cond}}$ as K,V. 3) Strided conv or upsampling for resolution changes. 4) Skip-connections between matching resolutions. A time-step embedding $\tau (t)\in R^{D}$ (sinusoidal or learned) is added to the ResBlock outputs to condition on diffusion step *t*.

**Table 7:** Model Hyperparameters for Spatia

Component	Parameter	Value
*Gene Processing Module*		
Gene Encoder	Embedding Dimension	256
	Vocabulary Size (Genes)	23122
Expression Encoder	Output Dimension	256
	Dropout	0.1
*Core Transformer*		
Flash Transformer Enc.	Number of Blocks	8
	Hidden Size (d_model)	256
	MLP Intermediate Size	1024
	Dropout	0.1
*Image Processing Module (ViTMAE)*		
ViT Encoder	Hidden Size	768
	Number of Layers	12
	Patch Size	16x16
	MLP Intermediate Size	3072
ViT Decoder	Hidden Size	512
	Number of Layers	8
	MLP Intermediate Size	2048
*Fusion Layers*		
Image Fusion Layer	Dimension	768
	Dropout	0.1
Expression Fusion Layer	Dimension	256
	Dropout	0.1
*Output Decoders*		
Expression Decoder	Hidden Dimension	256
	Dropout	0.1

#### C.1.2 Training Procedure

We freeze both the VAE and the fusion encoder $F_{\mathrm{fusion}}$, and train only the U-Net $\epsilon_{\omega }$ to predict noise in the VAE latent. We sample 3M cell-gene pairs $\left\{\left(C_{i},g_{i}\right)\right\}$ from MIST-C. Then precompute and cache latents $x_{i}=E_{\mathrm{diff}}\left(C_{i}\right)$ and embeddings $z_{i}$. The noise prediction loss is

$$\overset{ˆ}{\epsilon }=\epsilon_{\omega }\left(x_{i,t},t,z_{i}\right),\;{ℒ}_{diff}=E_{i,t,\epsilon }\left[\|\epsilon -\overset{ˆ}{\epsilon }{\|}_{2}^{2}\right].$$

where t∼Uniform{1,…,T}, and ϵ∼𝒩(0,I),

We use adamW with initial lr = 10^−4^, cosine decay, and 500-step warm-up. Train with 256 batch size and 25,000 steps.

#### C.1.3 Inference-Only Token Merging

We follow a three-step paradigm: 1) Token Importance Scoring. 2) Multi-directional Token Pairing via attention-based similarity. 3) Token Fusion.

We first partition the token sequence of length N based on token importance, using classifier-free guidance (CFG) [23] as an importance indicator. We then measure pairwise token similarities using the attention module’s query-key mechanism. Let ${\left\{x_{i}\right\}}_{i=1}^{N}$ denote the input tokens to a given Transformer layer, and $q\left(x_{i}\right),\;k\left(x_{j}\right)\in R^{d}$ represent the learned query and key projections of tokens $x_{i}$ and $x_{j}$. We define the similarity between two tokens as the scaled dot-product of their projections:

(12)
$$s_{ij}=\frac{q{\left(x_{i}\right)}^{⊤}k\left(x_{j}\right)}{\left\|q\left(x_{i}\right)\right\|\left\|k\left(x_{j}\right)\right\|},$$

which corresponds to the cosine similarity in the query-key space. Based on this mechanismm we then compute the pair similarity scores between anchor tokens and all tokens:

(13)
$${\tilde{s}}_{ij}=\left\{\begin{array}{ll} s_{ij}, & \mathrm{if}\;s_{ij}\geq \kappa ,\\ 0, & \mathrm{otherwise}, \end{array}\right.$$

where κ is a similarity threshold and $x_{i}\in 𝒮$ is an anchor token. This filtering step removes weakly related token pairs, mitigating the influence of noise or irrelevant associations. Unlike previous merging methods that enforce many-to-one pairing, our design allows overlaps, enabling a token to appear in multiple highly similar pairs. We evaluate the token Merging effectiveness by comparing the performance and generation speed with no merging. We are able to achieve 37% generation speedup with no performance loss.
